# Supplementary figures and images for: Bioactive Compounds and Antioxidant Properties of Wild Rocket (Diplotaxis Tenuifolia L.) Grown under Different Plastic Films and with Different UV-B Radiation Postharvest Treatments
Source: Foods. 2022 Dec 17;11(24):4093. doi: 10.3390/foods11244093 (PMC9778044; doi:10.3390/foods11244093)

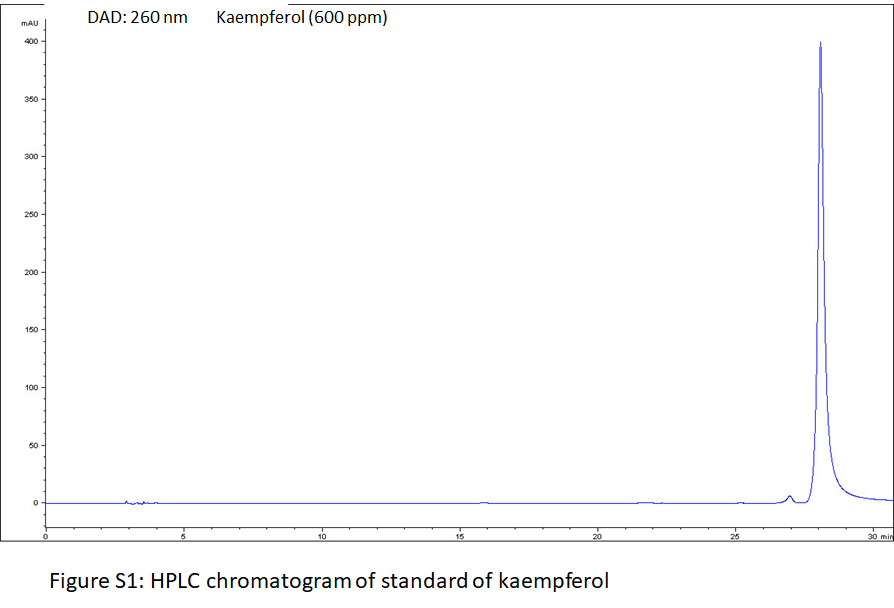

Supplement: Supplementary file 1 [file foods-11-04093-s001.zip › S1 con did.jpg]

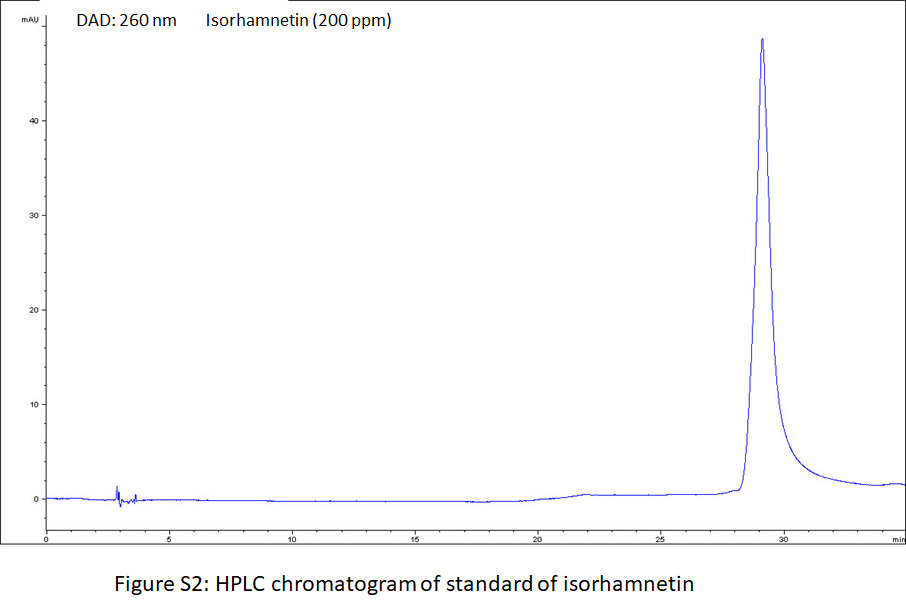

Supplement: Supplementary file 1 [file foods-11-04093-s001.zip › S2 con did.jpg]

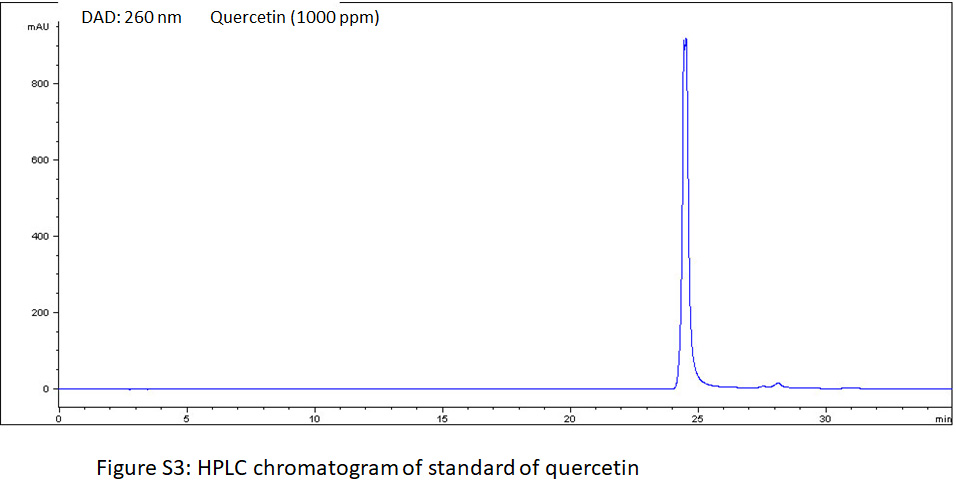

Supplement: Supplementary file 1 [file foods-11-04093-s001.zip › S3 con did.jpg]

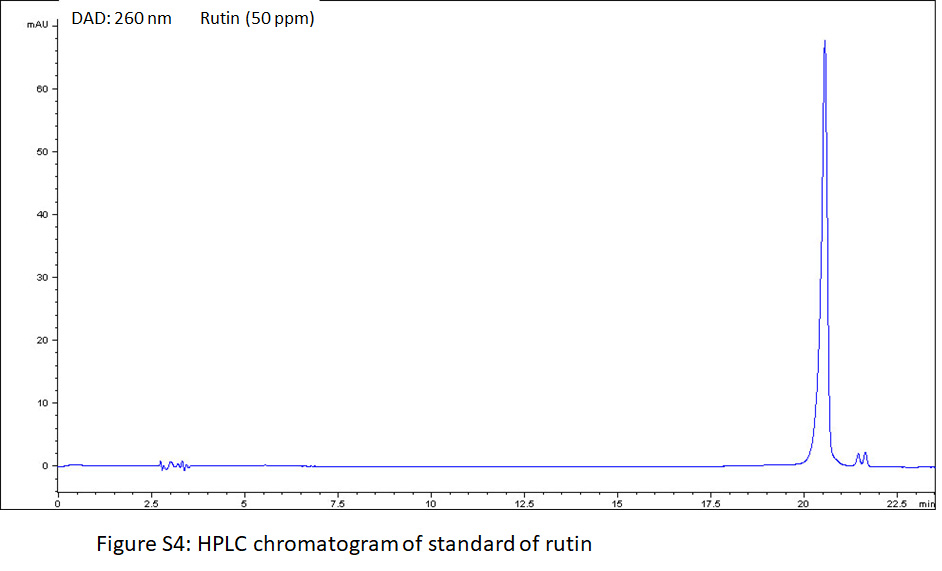

Supplement: Supplementary file 1 [file foods-11-04093-s001.zip › S4 con did.jpg]

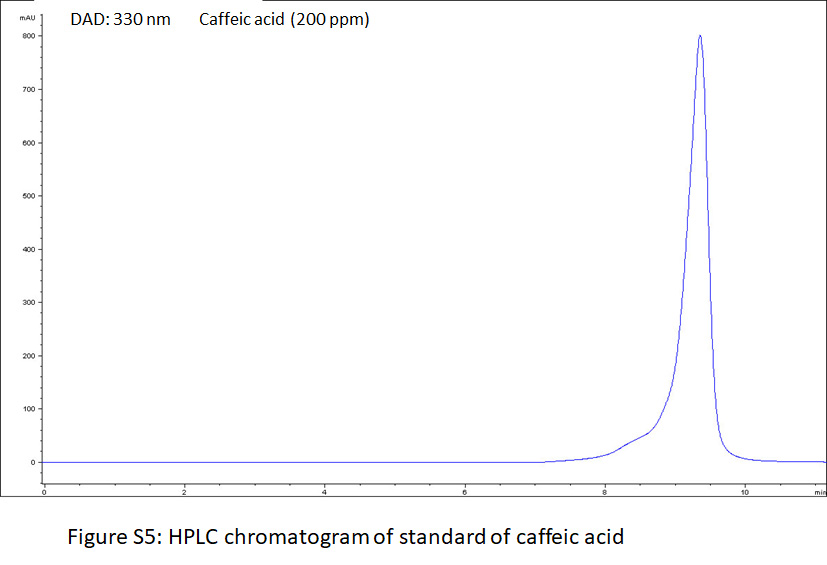

Supplement: Supplementary file 1 [file foods-11-04093-s001.zip › S5 con did.jpg]

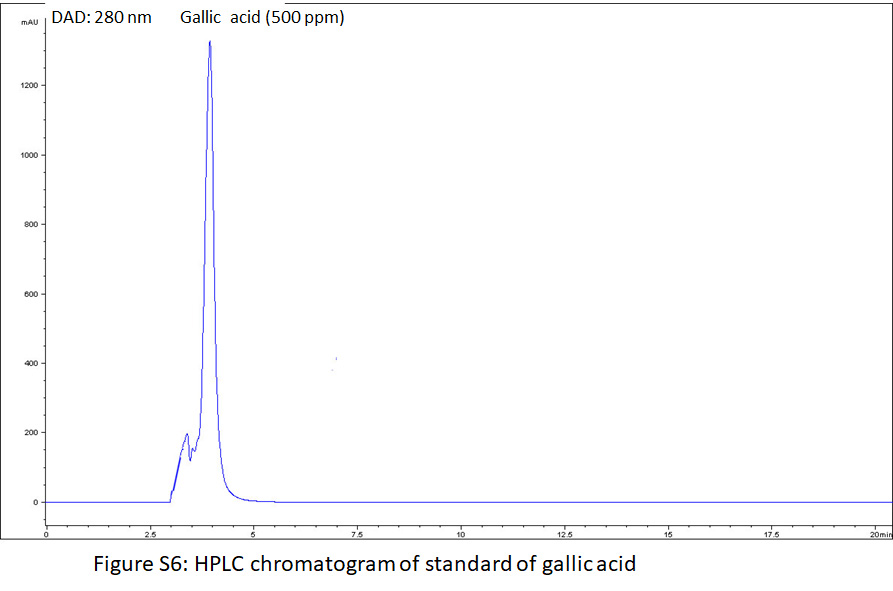

Supplement: Supplementary file 1 [file foods-11-04093-s001.zip › S6 con did.jpg]

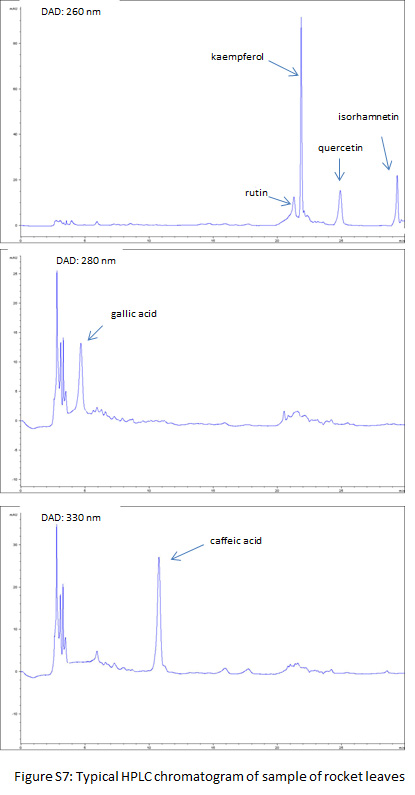

Supplement: Supplementary file 1 [file foods-11-04093-s001.zip › S7 con did.jpg]
